# Effect of proportional assist ventilation plus versus pressure support ventilation on successful weaning in critically ill adults: a systematic review, meta-analysis, and trial sequential analysis

**DOI:** 10.3389/fmed.2026.1775614

**Published:** 2026-02-25

**Authors:** Yifei Wang, Xiang Da, Xin Kang, Linlin Liu, Rui Zhao, Zi Zhu, Xinni Li, Yike Zhang, Conghui Wang, Jiehua Deng

**Affiliations:** 1 The Second Affiliated Hospital of Guilin Medical University, Guilin, Guangxi, China; 2 Jining Medical University, Jining, Shandong, China; 3 College of Health, Medicine and Wellbeing, The University of Newcastle, Newcastle, NSW, Australia; 4 School of Public Health, Sun Yat-sen University, Guangzhou, Guangdong, China; 5 Youyang Tujia and Miao Autonomous County People's Hospital, Chongqing, China; 6 Ganxi Vocational College of Science and Technology, Xinyu, Jiangxi, China

**Keywords:** critically ill adults, mechanical ventilation weaning, meta-analysis, pressure support ventilation (PSV), proportional assist ventilation plus

## Abstract

**Background:**

Proportional assist ventilation plus (PAV+) and pressure support ventilation (PSV) are commonly employed ventilatory modes during the weaning process from mechanical ventilation in critically ill adult patients. Proportional assist ventilation plus delivers assistance proportional to the patient’s inspiratory effort, thereby enhancing patient–ventilator synchrony and reducing the work of breathing. However, the efficacy of proportional assist ventilation in facilitating successful weaning remains a matter of debate.

**Methods:**

A comprehensive search was conducted in CENTRAL, PubMed, MEDLINE, Web of Science, Embase, and ClinicalTrials.gov from inception to October 1, 2025 We included randomized controlled trials comparing proportional assist ventilation plus with pressure support ventilation in adult patients (≥18 years) who underwent invasive mechanical ventilation for at least 24 h prior to weaning. The primary outcome was the rate of successful weaning, while secondary outcomes included reintubation rate, ICU and hospital mortality, ICU length of stay and duration of weaning. Trial sequential analysis and subgroup analyses based on clinical intent (spontaneous breathing trial vs. continuous weaning) were integrated to enhance the robustness of the findings.

**Results:**

In seven RCTs (*n* = 1, 214), proportional assist ventilation plus improved weaning success (RR = 1.12, 95% CI: 1.02–1.23). Consistently, this result remained significant in sensitivity analyses excluding spontaneous breathing trial studies. No significant differences were observed in reintubation, mortality, weaning duration, or ICU length of stay.

**Conclusion:**

Proportional assist ventilation plus improved weaning success compared with pressure support ventilation. This benefit remained robust in sensitivity analyses excluding spontaneous breathing trial studies. These findings suggest Proportional assist ventilation plus is a promising weaning mode, but further research is needed to optimize its implementation.

**Systematic review registration:**

PROSPERO, CRD420251170692.

## Introduction

Endotracheal intubation is highly prevalent among critically ill patients, with reported incidence rates of up to 60% (1). Each year, more than 13–20 million patients worldwide receive invasive mechanical ventilation (2). Although mechanical ventilation is essential for the management of critical illness, approximately 36.9% of patients experience difficulties during weaning and 16.5% experience prolonged weaning duration (3). Delayed weaning increases the risk of ventilator-associated pneumonia and diaphragmatic dysfunction, whereas premature extubation is associated with higher rates of reintubation, aspiration, and mortality (4).

Pressure support ventilation is currently the most commonly used partial-support mode, owing to its simplicity and widespread applicability, and it can effectively reduce the work of breathing (5). However, fixed-level pressure support ventilation cannot dynamically adjust to the patient’s respiratory effort, which may lead to patient–ventilator asynchrony and excessive ventilatory assistance (4, 5). In contrast, proportional assist ventilation plus, an advanced mode that automatically measures respiratory system compliance and resistance, modulates support in real time to align mechanical assistance with the patient’s spontaneous effort. This improves patient–ventilator synchrony, protects respiratory muscles, and may reduce sedation requirements (5). Selecting the optimal ventilation mode to facilitate safe and effective weaning remains a critical challenge in intensive care medicine.

Previous a meta-analysis (6) has suggested that proportional assist ventilation plus may increase the likelihood of successful weaning, reduce reintubation rates, and shorten ICU length of stay compared with pressure support ventilation. However, the trial sequential analysis acknowledges that the limited sample size restricts the reliability of the statistical outcomes. Moreover, a recent large multicenter randomized controlled trial including 573 patients reported no significant differences in primary clinical outcomes between proportional assist ventilation plus and pressure support ventilation groups, highlighting ongoing uncertainty in this field.

To address these limitations, the present study incorporated a newly published randomized controlled trial (6) and conducted an updated systematic review and meta-analysis. We integrated trial sequential analysis and subgroup analyses based on clinical intent (spontaneous breathing trial vs. continuous weaning) to evaluate the efficacy of proportional assist ventilation plus versus pressure support ventilation in the weaning process of adult patients receiving mechanical ventilation. The findings aim to provide more robust evidence to guide standardized, evidence-based weaning strategies in critically ill patients.

## Methods

### Protocol and guidance

This systematic review and meta-analysis was performed in accordance with the Preferred Reporting Items for Systematic Reviews and Meta-Analyses (PRISMA) guidelines (7). The review protocol was prospectively registered in the International Prospective Register of Systematic Reviews (PROSPERO; registration number CRD420251170692). All procedures, including study selection, data extraction, and quality assessment, were performed following the predefined protocol to ensure methodological transparency and reproducibility.

### Information sources and search strategy

A comprehensive search was conducted in CENTRAL, PubMed, MEDLINE, Web of Science, Embase, and ClinicalTrials.gov from inception to October 1, 2025. Preprints and online-first publications were included. Additionally, reference lists of eligible studies and related systematic reviews were screened, conference abstracts and gray literature were evaluated, and experts were consulted to ensure search completeness. No language or geographic restrictions were imposed. Two reviewers (YW and XD) independently screened studies in a blinded manner, with disagreements resolved by discussion or adjudication by a third reviewer (JD). Studies were included if their titles, abstracts, indexing terms, or keywords contained all of the following: “respiratory failure” “proportional assist ventilation” “pressure support ventilation” “mechanical ventilation weaning” “spontaneous breathing trial” and “randomized controlled trial.”

### Eligibility criteria

Studies were selected based on the PICOS framework as follows:

Population (P): The studies included in this review focused on adult critically ill patients aged 18 years or older who developed respiratory failure due to various causes, received invasive mechanical ventilation for at least 24 h, and were in the weaning phase. Studies involving neonates, children, or patients who were directly extubated to noninvasive ventilation were excluded.
Intervention (I): The intervention of interest was proportional assist ventilation, in which the level of pressure support is automatically adjusted according to the patient’s inspiratory flow and tidal volume to improve patient–ventilator synchrony and reduce the work of breathing.
Comparator (C): The comparator was pressure support ventilation, a conventional weaning mode in which fixed-level pressure support is applied during spontaneous inspiration.
Outcomes (O): The primary outcome was the rate of successful weaning, defined as the ability to maintain spontaneous breathing without invasive mechanical ventilation support for at least 48 h following extubation (or disconnection from the ventilator) (8). We accepted the definitions provided by the original trial authors if they aligned with these general criteria. Trials measuring success solely as passing a spontaneous breathing trial without follow-up were categorized separately in subgroup analyses. Secondary outcomes included reintubation rate, ICU mortality, hospital mortality, ICU length of stay and duration of weaning.
Study design (S): Only randomized controlled trials were eligible for inclusion in this review.

### Study selection

Two reviewers (YW and XD) independently screened the titles and abstracts of all records identified through the systematic search. Following the initial screening, the same reviewers performed a full-text review of the selected articles to determine eligibility. Any discrepancies between the reviewers were resolved through discussion to minimize potential bias. If consensus could not be reached, a third reviewer (JD) adjudicated the disagreement and made the final decision.

### Data extraction

Data were independently extracted by two reviewers (YW and XD) using a standardized form capturing study characteristics and participant information. In cases of missing or unclear data, we attempted to contact the corresponding authors of the original studies for clarification. A third reviewer (JD) checked all entries for accuracy and completeness. Any discrepancies during the data extraction process were resolved through discussion between the two reviewers, or by consulting the third reviewer (JD) to reach a consensus.

### Risk of bias assessment

**Figure 1:**
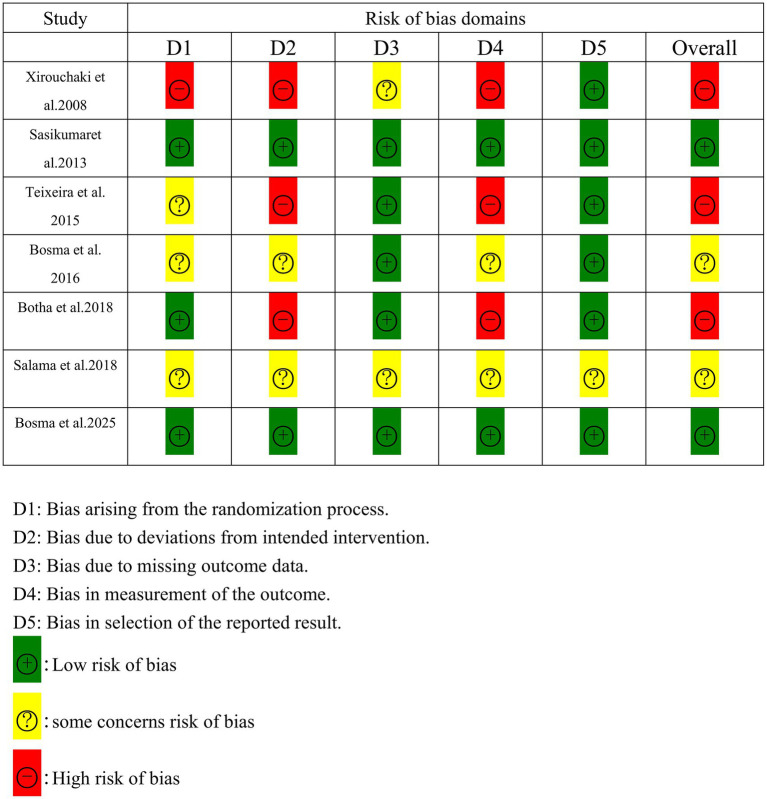
Risk of bias summary.

Two reviewers (YW and XD) independently assessed the risk of bias using the Cochrane Risk of Bias 2 (ROB 2) tool (9), which evaluates methodological quality across five domains: the randomization process, deviations from intended interventions, missing outcome data, outcome measurement, and selection of the reported result. Studies were judged as high risk if ≥1 domain was rated “high” low risk if all domains were “low,” and unclear risk if any domain was rated as “some concerns.” Any discrepancies regarding the risk of bias assessment were resolved through discussion or by consulting a third reviewer (JD) to reach a consensus (Figure 1).

### Confidence of evidence

Two reviewers (YW and XD) independently assessed the certainty of evidence using the GRADE approach (10), which evaluates outcomes across five domains: study design, risk of bias, inconsistency, imprecision, and indirectness. Evidence was graded as high, moderate, low, or very low. Any disagreements were resolved through discussion or adjudicated by a third reviewer (JD).

### Data analysis

All statistical analyses followed a pre-specified protocol and were performed using Review Manager (RevMan, version 5.4.1; Cochrane Collaboration). A random-effects model was applied to account for between-study heterogeneity. Odds ratios (ORs) with 95% confidence intervals (CIs) were calculated for dichotomous outcomes, and mean differences (MDs) for continuous outcomes, depending on the measurement scale. Statistical significance (11) was defined as a two-sided *p*-value < 0.05. Heterogeneity (12, 13) was assessed using the I^2^ statistic, interpreted as low (<25%), moderate (25–50%), substantial (50–75%), or considerable (>75%). When substantial heterogeneity was observed, sensitivity analyses were performed when feasible. Trial sequential analysis was conducted to control for cumulative type I error and to estimate the required information size. All analyses adhered to the Cochrane Handbook for Systematic Reviews of Interventions.

### Trial sequential analysis

This study employed trial sequential analysis to estimate the required sample size for the meta-analysis and to determine whether the existing data provided sufficient statistical power to support the conclusions. By controlling the risks of type I and type II errors, trial sequential analysis helps ascertain whether additional trials are necessary to confirm the effect size. The required information size was calculated and the statistical power of the cumulative data was evaluated based on the following pre specified parameters: a significance level (*α*) of 0.05, statistical power (1 − *β*) of 0.80, a control group event rate of 43.9%, an expected intervention group event rate of 52.7%, and a pre-specified relative risk reduction threshold of 20%. This 20% relative risk reduction threshold was informed by prior meta-analysis (6) representing the minimum clinically important difference and establishing a conservative, rigorous criterion for statistical significance aimed at mitigating the risk of type I error. Ultimately, by comparing the actual cumulative sample size with the required information size, we confirmed that the accrued sample size had reached the required information size, indicating that the findings are not compromised by sample size insufficiency and that the results are robust and reliable.

### Sensitivity analysis

To further assess the robustness of our findings, several sensitivity analyses were performed: (1) a fixed-effects model was applied to evaluate the impact of the statistical model choice; (2) a leave-one-out analysis was performed, in which each study was sequentially removed to examine its influence on the overall effect estimates; and (3) studies categorized as the ‘SBT phase’ were excluded to specifically evaluate the robustness of the primary outcome within the context of the continuous weaning process.

### Subgroup analysis

To further investigate potential sources of clinical heterogeneity in the effectiveness of proportional assist ventilation plus (PAV+) for improving weaning success, we conducted a subgroup analysis of the primary outcome based on the clinical intent—specifically, the mode of application—of the study protocols. Studies were categorized as follows: Spontaneous breathing trial phase: In these studies, either proportional assist ventilation plus or pressure support ventilation (PSV) was applied only during a short, predefined test period (typically 30–120 min). The primary intent was to use the mode as a predictive tool to assess whether the patient was ready for immediate extubation. Accordingly, weaning success was defined as passing this brief trial and proceeding to extubation; Continuous weaning: In these studies, proportional assist ventilation plus (or pressure support ventilation) was applied throughout the active weaning phase—that is, from the initiation of weaning efforts until the decision to discontinue mechanical ventilation (either by extubation or weaning completion). Here, the intent was to employ the mode as a primary therapeutic means to actively facilitate and gradually reduce ventilatory support over a period of hours to days.

## Results

Table 1 summarizes the key characteristics of the included studies. All participants were adult patients with critical respiratory failure who required invasive mechanical ventilation. The randomized controlled trials included in this meta-analysis were published between 2008 and 2025, with sample sizes ranging from 23 to 573 participants per study. The studies varied in terms of their target populations and clinical settings. Two studies (14, 15)included patients from mixed intensive care units, encompassing those with trauma, sepsis, acute respiratory distress syndrome, and postoperative respiratory failure. A study (15) specifically focused on patients with traumatic brain injury and another (16) included critically ill patients who had been on mechanical ventilation for at least 48 h and were capable of tolerating partial spontaneous breathing. Although all the studies (14–20) aimed to evaluate critically ill patients in the weaning phase of mechanical ventilation, there was considerable heterogeneity across the studies.

**Table 1:** Characteristics of included studies.

Authors	Year	Design	Patient number	Age year	Male (%)	Mean MV (days)	Mean PS	Main disease	Intervention 1	Intervention 2
Xirouchaki et al.	2008	RCT	208	60.9	66.3	4.0	APACHEII, 15.5	Acute lung injury, Acute respiratory distress syndrome, COPD and other	PAV+: initial assist60-80%, reduced by10-20% per hour. Extubation was performed at 10-20%assist.	PSV: initial PIP 20–25 cmH_2_O, reduced by 2–5 cmH_2_Oper hour. Extubation was performed at PS 10–12 cmH_2_O.
Sasikumar et al.	2013	RCT	23	48.6	69.6	NR	APACHE II, 20.7	Acute lung injury, Acute respiratory distress syndrome, Sepsis and other	PAV+: the protocol for PAV + setting was not available.	PSV: the protocol for PSV setting was not available.
Teixeira et al.	2015	RCT	160	44.5	65.6	6.6	APACHE II, 22.7	COPD, Congestive heart failure, Traumatic brain injury and other	PAV+: initial assist and the method for reduction were not available. Extubation was performed at 40% assist.	PSV: pressure support with 7 cmH_2_O. No reduction method was mentioned. The timing for extubation was not available.
Bosma et al.	2016	RCT	50	64.8	50.0	5.8	APACHE II, 26.5	COPD, Pneumonia, Cardiac arrest and other	PAV+: initial assist 70%, reduced by the judgment of a respiratory therapist every 2 to 3 h.	PSV: initial PS 15 cmH_2_O, reduced by the judgment of a respiratory therapist every 2 to 3 h.
Botha et al.	2018	RCT	50	63.2	59.2	3.4	APACHE III, 76.7; SASP, 45.5	Respiratory, Cardiac, Neurological and other	PAV+: initial assist 70%, reduced by 10%. Extubation was performed at 30% assist.	PSV: initial PS was set at patient’s need; the reduction method was not mentioned. Extubation was performed at PS 10 cmH_2_O.
Salama et al.	2018	RCT	150	NA	NA	NA	NA	NA	PAV+	PSV
Bosma et al.	2025	RCT	573	62.1	59.2	4.9	APACHE III: 80.3	Cardiovascular conditions, Sepsis, Gastrointestinal conditions and other	PAV+: Gain adjusted to keep peak respiratory-muscle pressure 5–10 cm H₂O (mean 65 ± 10%)	PSV: Pressure support adjusted to maintain respiratory rate 12–35 breaths/min and tidal volume 5–10 mL/kg (mean initial PS 10.8 ± 3.0 cm H₂O).

RCT = randomized controlled trial; MV = mechanical ventilation; PS = severity score (e.g., APACHE, SAPS); PAV+ = proportional assist ventilation plus; PSV = pressure support ventilation; NR = not reported; NA = not applicable; COPD = chronic obstructive pulmonary disease.

**Figure 2:**
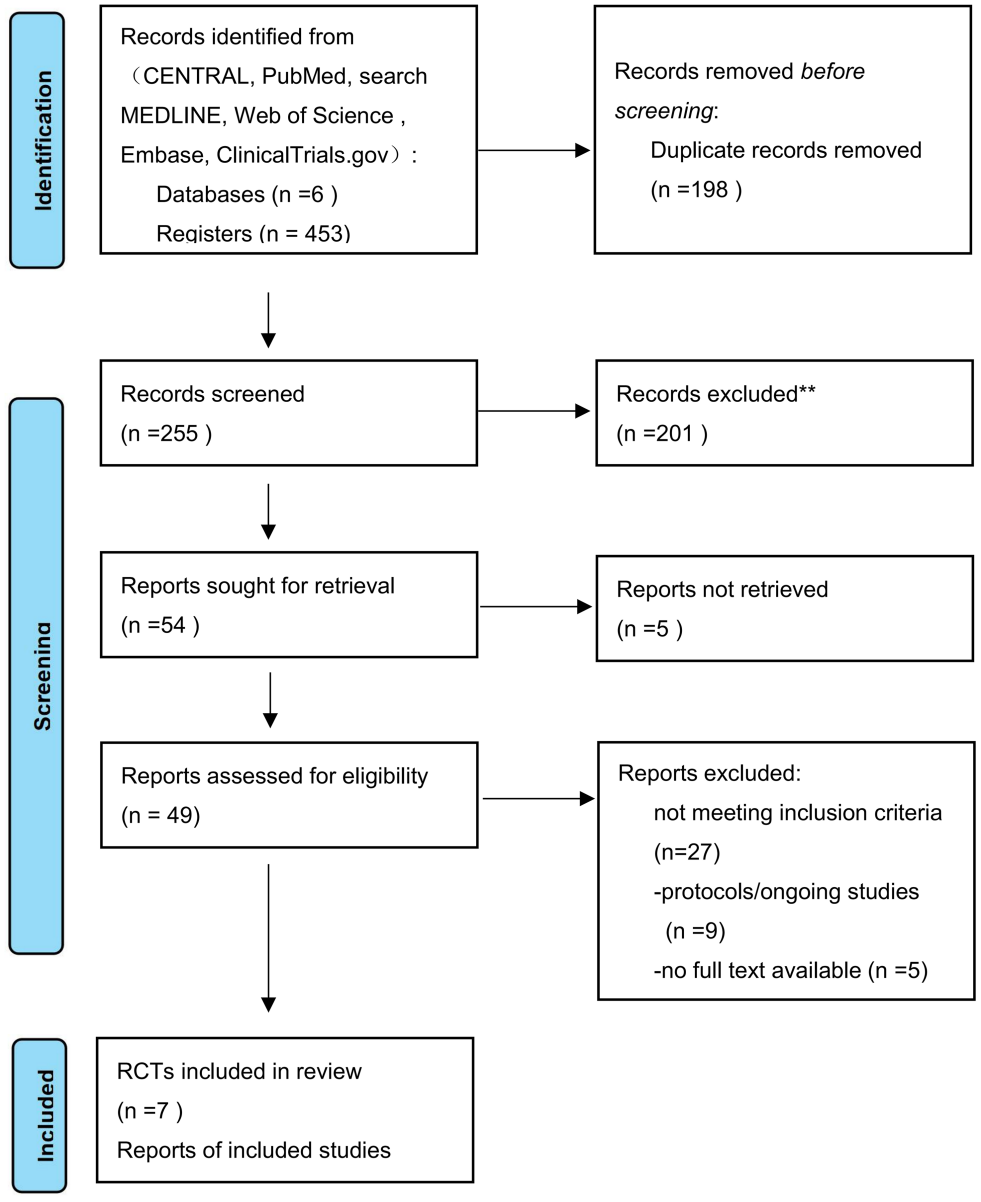
Flow chart of enrollment.

**Figure 3:**
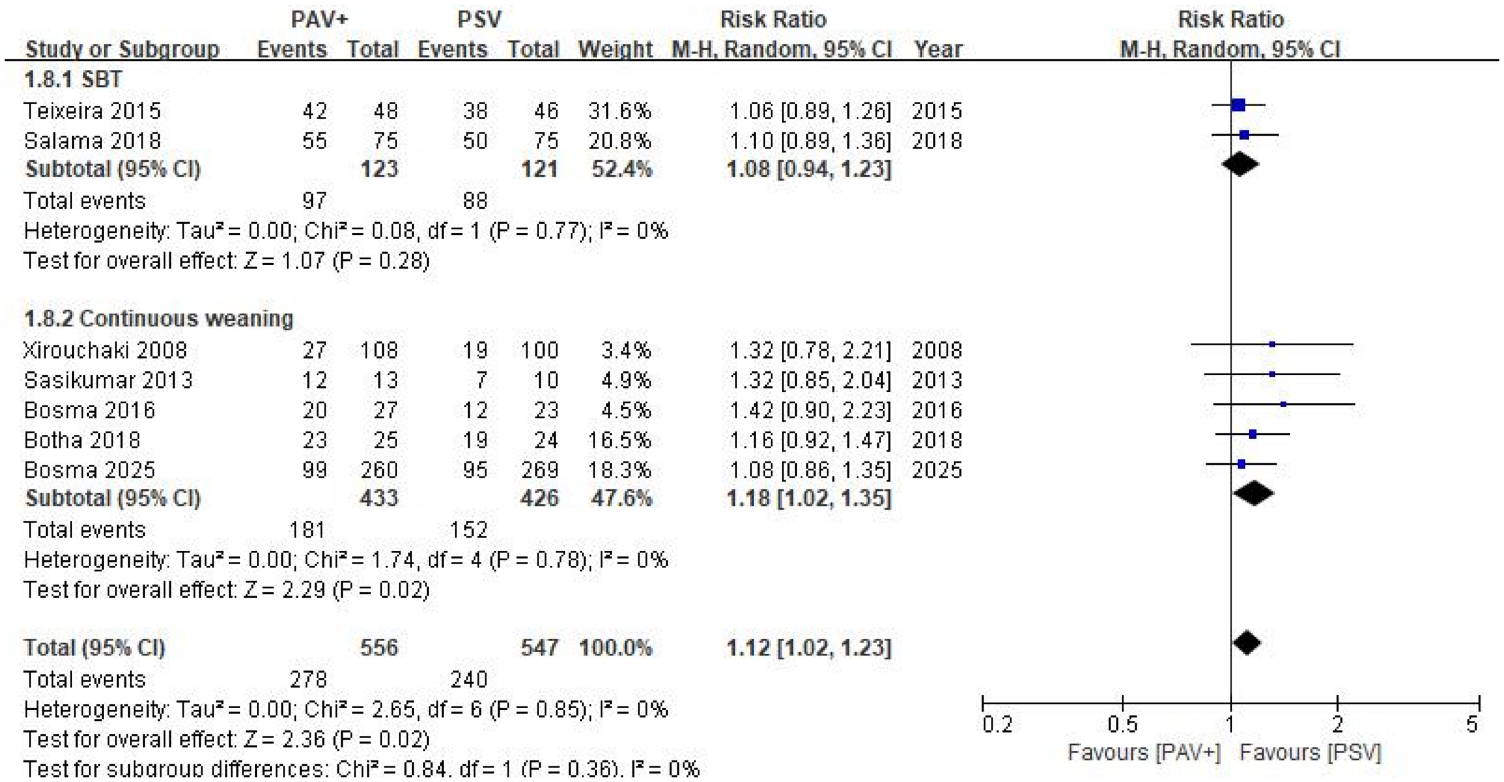
Forest plot of the primary outcome: weaning success rate comparing PAV + versus PSV.

This meta-analysis included a total of 7 (14–20) randomized controlled trials, with 1,214 critically ill patients receiving invasive mechanical ventilation (Figure 2). Of these, 556 patients were in the proportional assist ventilation plus group, with a weaning success rate of 50.0%, while 547 patients were in the pressure support ventilation group, with a weaning success rate of 43.9%. The pooled analysis revealed that the weaning success rate in the proportional assist ventilation plus group was higher than that in the pressure support ventilation group (RR = 1.12, 95% CI: 1.02–1.23; Figure 3). Subgroup analysis based on clinical intent indicated a similar trend in both SBT (RR = 1.08, 95% CI: 0.94–1.23) and Continuous weaning (RR = 1.18, 95% CI: 1.02–1.35) groups, with no significant interaction observed between subgroups (P for interaction = 0.36). Trial sequential analysis of weaning success suggested that a sample size of 1,013 participants would be required to ensure sufficient statistical power, indicating that our sample size was adequate to detect meaningful differences. To assess the robustness of these findings, several sensitivity analyses were performed (Table 2): (1) application of a fixed-effects model; (2) leave-one-out analysis; and (3) exclusion of studies categorized as the SBT phase (15, 16). Notably, after excluding the two SBT-related trials, the combined effect size for weaning success remained statistically significant (RR = 1.18, 95% CI: 1.02–1.35). This suggests that the overall positive effect of PAV + on weaning success is robust and not solely driven by studies conducted during the short-term SBT phase. The overall strength of evidence is moderate to low, with key findings focused on the short-term symptomatic benefits of the interventions (Table 3).

**Table 2:** Sensitivity analysis of meta-analysis.

Sensitivity analysis	No. patients (trials)	RR	95%CI
All trials	1,103 (7)	1.12	1.02, 1.23
Using fixed-effect models	1,103 (7)	1.13	1.01, 1.27
Excluding studies with SBT^1^	859 (5)	1.18	1.02, 1.35
Excluding studies published before^2^	872 (5)	1.11	1.00, 1.22
Excluding each randomized control trial in turn			
Excluding Xirouchaki et al. (14)	895 (6)	1.12	1.01, 1.23
Excluding Sasikumar et al. (16)	1,080 (6)	1.11	1.01, 1.23
Excluding Teixeira et al. (15)	1,009 (6)	1.15	1.03, 1.29
Excluding Bosma et al. (18)	1,053 (6)	1.11	1.01, 1.22
Excluding Botha et al. (19)	953 (6)	1.13	1.01, 1.26
Excluding Salama et al. (20)	1,054 (6)	1.11	1.00, 1.24
Excluding Bosma et al. (17)	574 (6)	1.13	1.02, 1.26

CI, confidence interval; RR, Risk Ratio.

The two studies by Teixeira et al. (15) and Salama et al. (20) applied the intervention modes (PAV + vs. PSV) solely during a short-term spontaneous breathing trial (SBT) to evaluate their predictive value for extubation outcome. As their design fundamentally differs from trials that applied the modes throughout the weaning process, they were excluded in this sensitivity analysis to ensure the analysis specifically addresses the effect of continuous mode application during continuous weaning.

Exclusion of studies published prior to 2015, to ensure that the synthesized evidence reflects contemporary standards of critical care, mechanical ventilation technology, and weaning protocols, thereby enhancing the clinical relevance of the conclusions to current practice.

**Table 3:** Summary of findings and strength of evidence.

Outcome	No. of patients (Trials)	Relative effect, Risk Ratio (95%MD/CI)	Absolute effect estimates (per 1,000)			Quality of the evidence
			PAV+	PSV	Difference	
The primary outcome						
Weaning success	1,103 (7)	1.12 (1.02, 1.23)	278	240	53 [9, 101]	Moderate
The secondary outcome						
Reintubation	908 (6)	0.87 (0.65, 1.17)	65	76	−22 [−59, 29]	Moderate
In-hospital mortality	974 (5)	0.90 (0.67, 1.22)	120	124	−26 [−85, 57]	Moderate
ICU mortality	974 (5)	0.90 (0.66, 1.23)	79	86	−18 [−61, 41]	Moderate
ICU length of stay (days)	789 (5)	0.15 (−1.13, 1.44)				Low
Duration of weaning (hours)	695 (4)	−0.01 (−1.27, 1.25)				Low

CI, confidence interval; RR, Risk Ratio; MD, mean difference.

Six studies (13–19) reported the reintubation rate. Among the 460 patients in the proportional assist ventilation plus group, 65 (14.1%) required reintubation, while 76 (17.0%) of 448 patients in the pressure support ventilation group were reintubated (Supplementary Figure S1). There was no significant difference between the two groups (RR = 0.87, 95% CI: 0.65–1.17). Furthermore, subgroup analysis based on clinical intent (SBT vs. Continuous weaning) demonstrated consistent results, with no significant difference observed between the SBT (RR = 0.72, 95% CI: 0.27–1.91) and Continuous weaning (RR = 0.89, 95% CI: 0.65–1.22) subgroups (P for interaction = 0.68). Five studies (6, 14, 15, 18, 19) assessed in-hospital mortality. In the proportional assist ventilation plus group, 120 out of 495 patients (24.2%) died, compared to 124 out of 479 patients (25.9%) in the pressure support ventilation group, with no significant difference between groups (RR = 0.90, 95% CI: 0.67–1.22) (Supplementary Figure S2). Subgroup analysis showed consistent results between the SBT (RR = 0.80, 95% CI: 0.26–2.44) and continuous weaning (RR = 0.89, 95% CI: 0.62–1.29) subgroups, with no significant interaction observed (P for interaction = 0.85). Five studies (14, 15, 17–19) reported ICU mortality (Supplementary Figure S3). In the proportional assist ventilation plus group, 79 out of 495 patients (16.0%) died, while 86 out of 479 patients (18.0%) in the pressure support ventilation group died, showing no significant difference (RR = 0.90, 95% CI: 0.66–1.23), Subgroup analysis showed no significant difference between the SBT (RR = 0.32, 95% CI: 0.01–7.65) and continuous weaning (RR = 0.88, 95% CI: 0.61–1.29) subgroups (P for interaction = 0.53). No significant differences were observed between proportional assist ventilation plus (PAV+) and pressure support ventilation (PSV) groups in terms of ICU length of stay (5 studies (15–19); MD = 0.15, 95% CI: −1.13 to 1.44; Supplementary Figure S4) or weaning duration (4 studies (16–19); MD = −0.01, 95% CI: −1.27 to 1.25; Supplementary Figure S5), with subgroup analyses based on clinical intent (SBT vs. Continuous weaning) confirming the consistency of these findings (P for interaction = 0.93).

## Discussion

This meta-analysis included 7 studies (14–20) involving 1,214 critically ill patients who received mechanical ventilation. Our findings demonstrate that, compared to pressure support ventilation, proportional assist ventilation plus significantly increased the weaning success rate. This result provides robust evidence supporting the application of proportional assist ventilation plus in critically ill patients, highlighting its potential advantages in facilitating weaning. Furthermore, sensitivity analysis indicated that this benefit remained statistically significant after excluding studies conducted during the short-term spontaneous breathing trial phase, suggesting that the efficacy of proportional assist ventilation plus is consistent across different clinical contexts.

Our study demonstrates that proportional assist ventilation plus significantly improves weaning success rates compared to pressure support ventilation, a finding consistent with previous meta-analyses. However, the meta-analyses acknowledged that small sample sizes could affect the reliability of the findings. A trial sequential analysis (5) suggested that 1,074 participants were needed for adequate statistical power, but only 634 were included, raising the risk of insufficient power. Furthermore, a recent large multi-center randomized controlled trial (6) involving 573 patients found no significant difference in outcomes between the proportional assist ventilation plus and pressure support ventilation groups, further highlighting the uncertainty in this field. In contrast, our meta-analysis incorporated more recent and larger randomized controlled trials, with a total of 1,103 participants—reaching the required information size. This substantial increase in sample size significantly enhanced the statistical power and reliability of our findings. Consequently, our results provide stronger evidence supporting the potential benefits of proportional assist ventilation in the weaning process for critically ill patients undergoing mechanical ventilation.

Proportional assist ventilation plus is an adaptive mode of mechanical ventilation that builds upon the concept of dynamic support by continuously and proportionally adjusting pressure support in response to the patient’s spontaneous inspiratory effort (24). This mechanism enhances patient-ventilator synchrony and interaction, which has been shown to improve weaning outcomes (21, 23). Although a previous meta analysis suggested potential benefits of proportional assist ventilation plus, its findings were limited by insufficient sample size (22). Recent expert consensus guidelines (25) similarly suggest that using proportional assist ventilation plus during initial spontaneous breathing trials may enhance extubation success and reduce ICU mortality. Crucially, while our meta-analysis confirms an overall benefit, the physiological basis of proportional assist ventilation plus suggests that clinical efficacy may be most pronounced in patients prone to asynchrony, particularly those with chronic obstructive pulmonary diseases. Conversely, caution is warranted in patients with unstable respiratory drive (e.g., severe central nervous system injury) or excessive air leaks, as proportional assist ventilation plus relies on adequate intrinsic effort to regulate proportional support. Despite these potential advantages, the widespread clinical implementation of proportional assist ventilation plus is hindered by the lack of simple, reliable tools for assessing patient-ventilator interaction (24). Consequently, larger-scale studies are required to further validate the long-term clinical utility of this mode.

We also acknowledge that our study has several limitations. First, the included studies involved critically ill ICU patients with various underlying conditions, such as cardiac or respiratory failure, neurological disorders, and trauma. The heterogeneity in these patient populations may introduce confounding effects, and caution is needed when interpreting the results. Second, the included studies did not specifically assess outcomes related to the reduction of sedative use or the incidence of delirium, both of which are crucial clinical targets in ICU care. Future research should further explore the potential impact of proportional assist ventilation plus on reducing sedative use, preventing delirium, and improving neurocognitive function.

## Conclusion

This meta-analysis demonstrates that proportional assist ventilation plus significantly improves weaning success rates, although it did not demonstrate significant benefits in terms of reintubation rates, mortality, or hospital length of stay. While proportional assist ventilation plus represents a promising physiological weaning strategy, its clinical implementation should be carefully evaluated based on individual patient characteristics. Further large-scale studies are needed to identify the optimal patient populations and timing for its application.

## Data Availability

The datasets presented in this study can be found in online repositories. The names of the repository/repositories and accession number(s) can be found in the article/Supplementary material.
