# Supplementary material for: Effect of proportional assist ventilation plus versus pressure support ventilation on successful weaning in critically ill adults: a systematic review, meta-analysis, and trial sequential analysis
Source: Front Med (Lausanne). 2026 Feb 25;13:1775614. doi: 10.3389/fmed.2026.1775614 (PMC12975930; doi:10.3389/fmed.2026.1775614)
Supplement: Supplementary file 1 [file Data_Sheet_1.docx]

**Pubmed search strategy to October, 2025**

#1 ("Weaning"[MeSH Terms] OR "Ventilator Weaning"[MeSH Terms] OR "weaning"[Title/Abstract] OR "spontaneous breathing trial"[Title/Abstract] OR "spontaneous breathing test"[Title/Abstract] OR "SBT"[Title/Abstract] OR "ventilator liberation"[Title/Abstract]) OR ("Respiration, Artificial"[MeSH Terms] OR "mechanical ventilation"[Title/Abstract] OR "ventilator"[Title/Abstract])

#2 ("Pressure Support"[Title/Abstract] OR "pressure support ventilation"[Title/Abstract] OR "PSV"[Title/Abstract] OR "Interactive Ventilatory Support"[MeSH Terms])

#3 ("Proportional Assist Ventilation"[Title/Abstract] OR "proportional assisted ventilation"[Title/Abstract] OR "PAV"[Title/Abstract] OR "PAV+"[Title/Abstract])

#4 #1 AND #2 AND #3

**Ovid search strategy to October, 2025**

#1

("weaning" OR "spontaneous breathing test" OR "spontaneous breathing trial" OR "sbt" OR "spontaneous breathing" OR "ventilator" OR "mechanical ventilation" OR "ventilation").mp.

#2

("pressure support" OR "pressure support mode" OR "pressure support ventilation").mp.

#3

("proportional assist ventilation" OR "proportional assist ventilator" OR "proportional assist" OR "proportional assisted ventilation" OR "PAV" OR "PAV+").mp.

#4

1 AND 2 AND 3

**Web of Science search strategy to October, 2025**

TS=("weaning" OR "spontaneous breathing trial" OR "spontaneous breathing test" OR "SBT" OR "ventilator liberation" OR "mechanical ventilation")

AND

TS=("pressure support" OR "pressure support ventilation" OR "PSV")

AND

TS=("proportional assist ventilation" OR "proportional assisted ventilation" OR "PAV" OR "PAV+")

**Embase search strategy to October, 2025**

1. 'weaning'/exp OR 'ventilator weaning'/exp OR 'spontaneous breathing trial'/exp OR 'ventilator liberation' OR'sbt':ti,ab

2. 'artificial ventilation'/exp OR 'mechanical ventilation'/exp

3. 1 OR 2

4. 'pressure support ventilation'/exp OR 'pressure support ventilation' OR 'pressure support'

5. 'proportional assist ventilation'/exp OR 'proportional assist ventilation' OR 'PAV':ti,ab OR 'PAV+'

6. 3 AND 4 AND 5

[**ClinicalTrials.gov**](https://clinicaltrials.gov/) **search strategy to October, 2025**

(weaning OR mechanical ventilation) AND (pressure support ventilation OR pressure support) AND (proportional assist ventilation OR PAV OR PAV+)

**MEDLINE search strategy to October, 2025**

1. exp Ventilator Weaning/ OR weaning.mp. OR spontaneous breathing test.mp. OR spontaneous breathing trial.mp. OR sbt.mp. OR ventilator liberation.mp.

2. exp Respiration, Artificial/ OR mechanical ventilation.mp. OR ventilator.mp.

3. 1 OR 2

4. pressure support.mp. OR pressure support ventilation.mp. OR PSV.ti,ab.

5. proportional assist ventilation.mp. OR proportional assisted ventilation.mp. OR PAV.ti,ab. OR PAV+.mp.

6. 3 AND 4 AND 5
