# Supplementary material for: Effect of proportional assist ventilation plus versus pressure support ventilation on successful weaning in critically ill adults: a systematic review, meta-analysis, and trial sequential analysis
Source: Front Med (Lausanne). 2026 Feb 25;13:1775614. doi: 10.3389/fmed.2026.1775614 (PMC12975930; doi:10.3389/fmed.2026.1775614)

**Supplement Information**

| **Figure S1**. Forest plot of reintubation rates comparing PAV+ versus PSV. |
| --- |
| **Figure S2**. Forest plot of in-hospital mortality comparing PAV+ versus PSV. |
| **Figure S3**. Forest plot of ICU mortality comparing PAV+ versus PSV. |
| **Figure S4**. Forest plot of ICU length of stay (days) comparing PAV+ versus PSV. |
| **Figure S5**. Forest plot of weaning duration (hours) comparing PAV+ versus PSV. |

**Figure S1**. Forest plot of reintubation rates comparing PAV+ versus PSV.


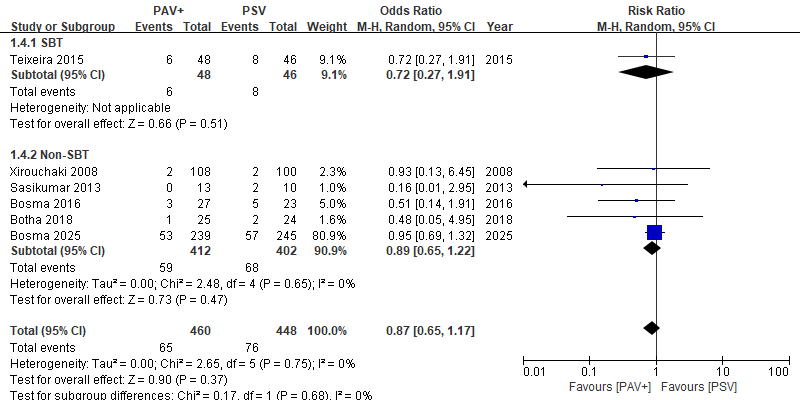


**Figure S2**. Forest plot of in-hospital mortality comparing PAV+ versus PSV.


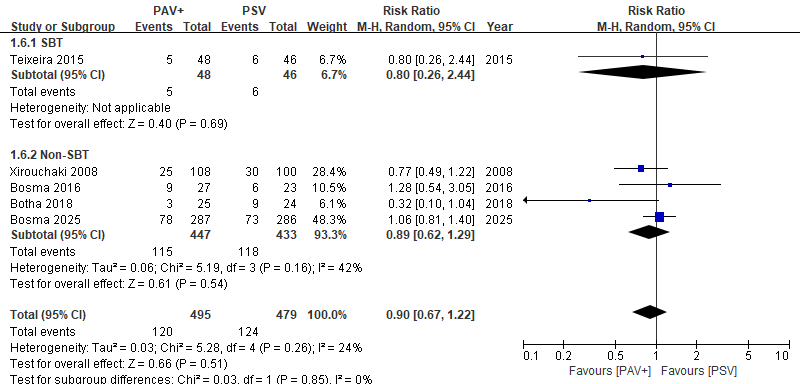


**Figure S3**. Forest plot of ICU mortality comparing PAV+ versus PSV.


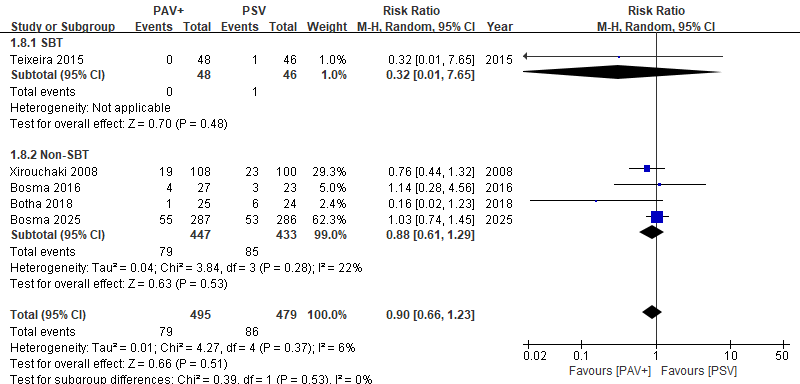


**Figure S4**. Forest plot of ICU length of stay (days) comparing PAV+ versus PSV.


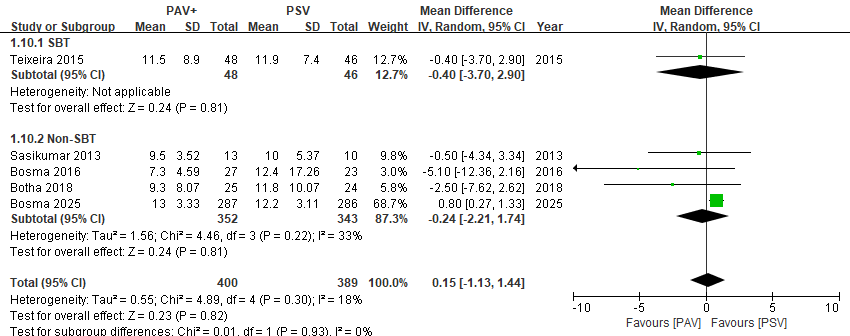


**Figure S5**. Forest plot of weaning duration (hours) comparing PAV+ versus PSV.


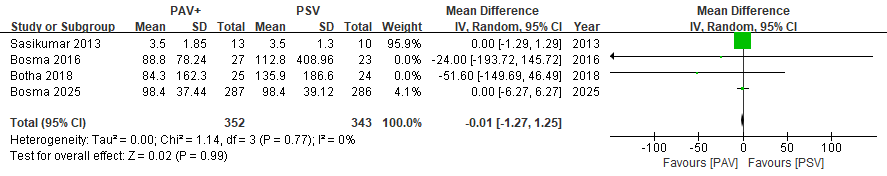

Supplement: Supplementary file 2 [file Data_Sheet_2.docx]
