# Supplementary figures and images for: Effect of proportional assist ventilation plus versus pressure support ventilation on successful weaning in critically ill adults: a systematic review, meta-analysis, and trial sequential analysis
Source: Front Med (Lausanne). 2026 Feb 25;13:1775614. doi: 10.3389/fmed.2026.1775614 (PMC12975930; doi:10.3389/fmed.2026.1775614)

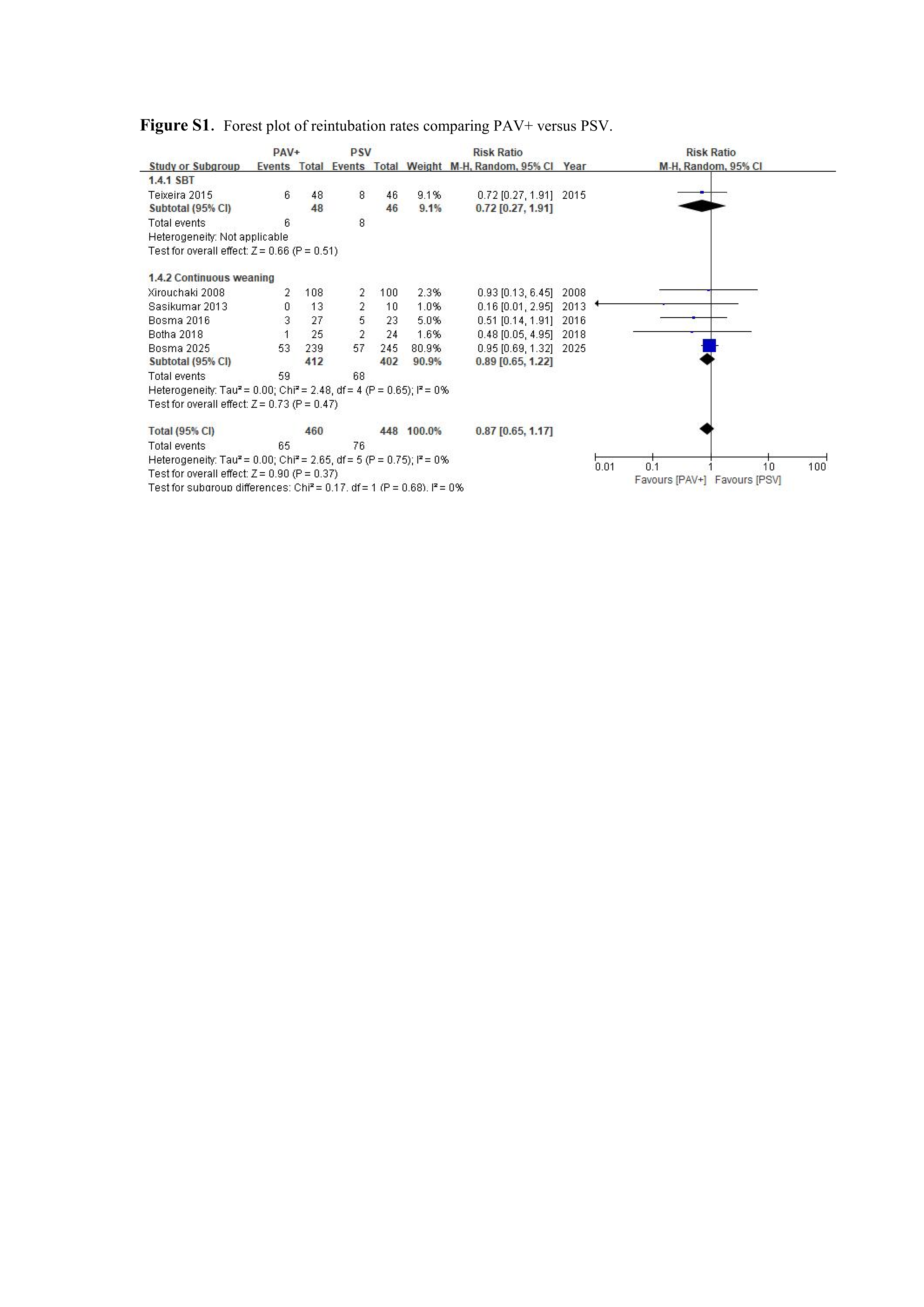

Supplement: Supplementary file 3 [file Image_1.png]

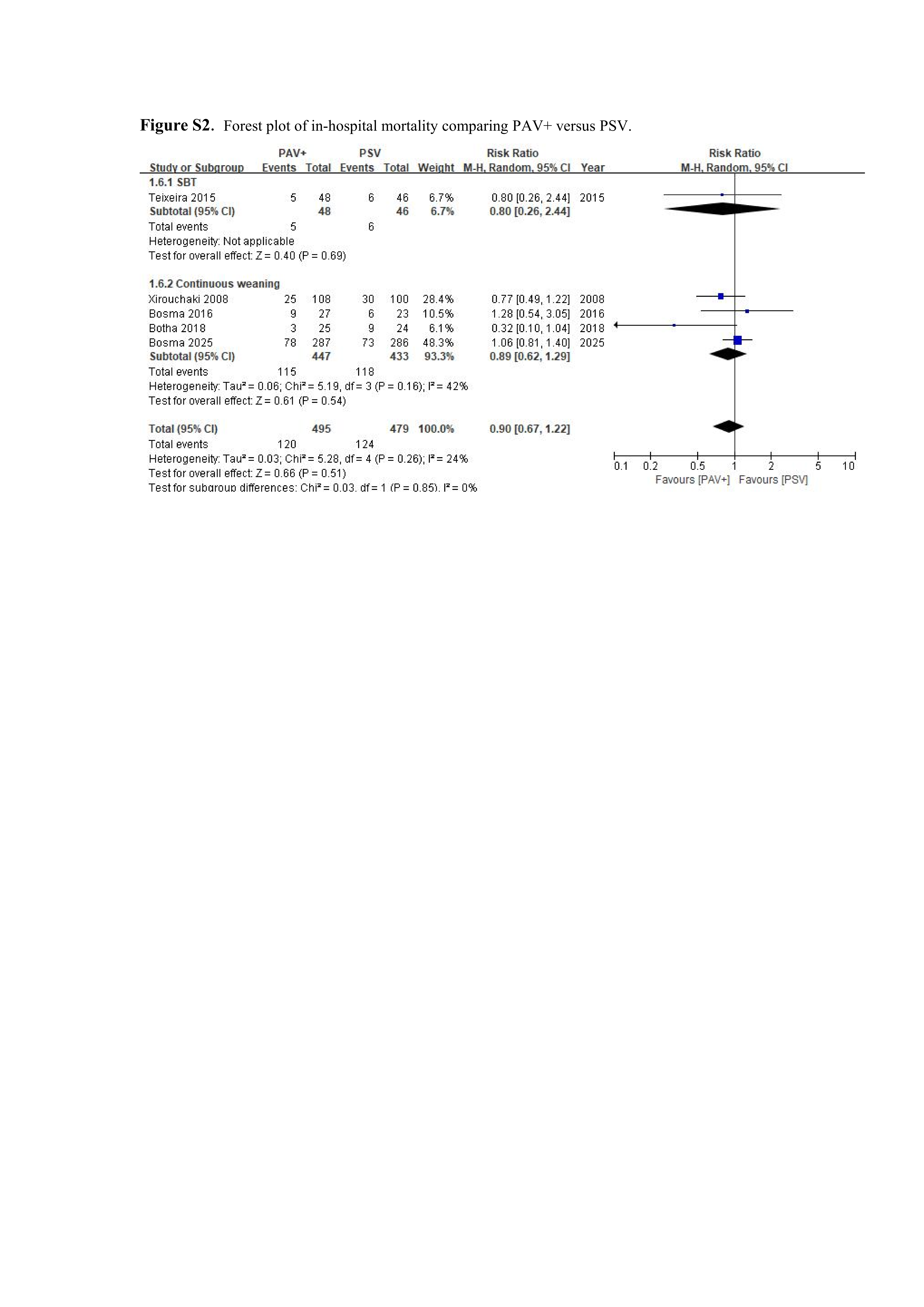

Supplement: Supplementary file 4 [file Image_2.png]

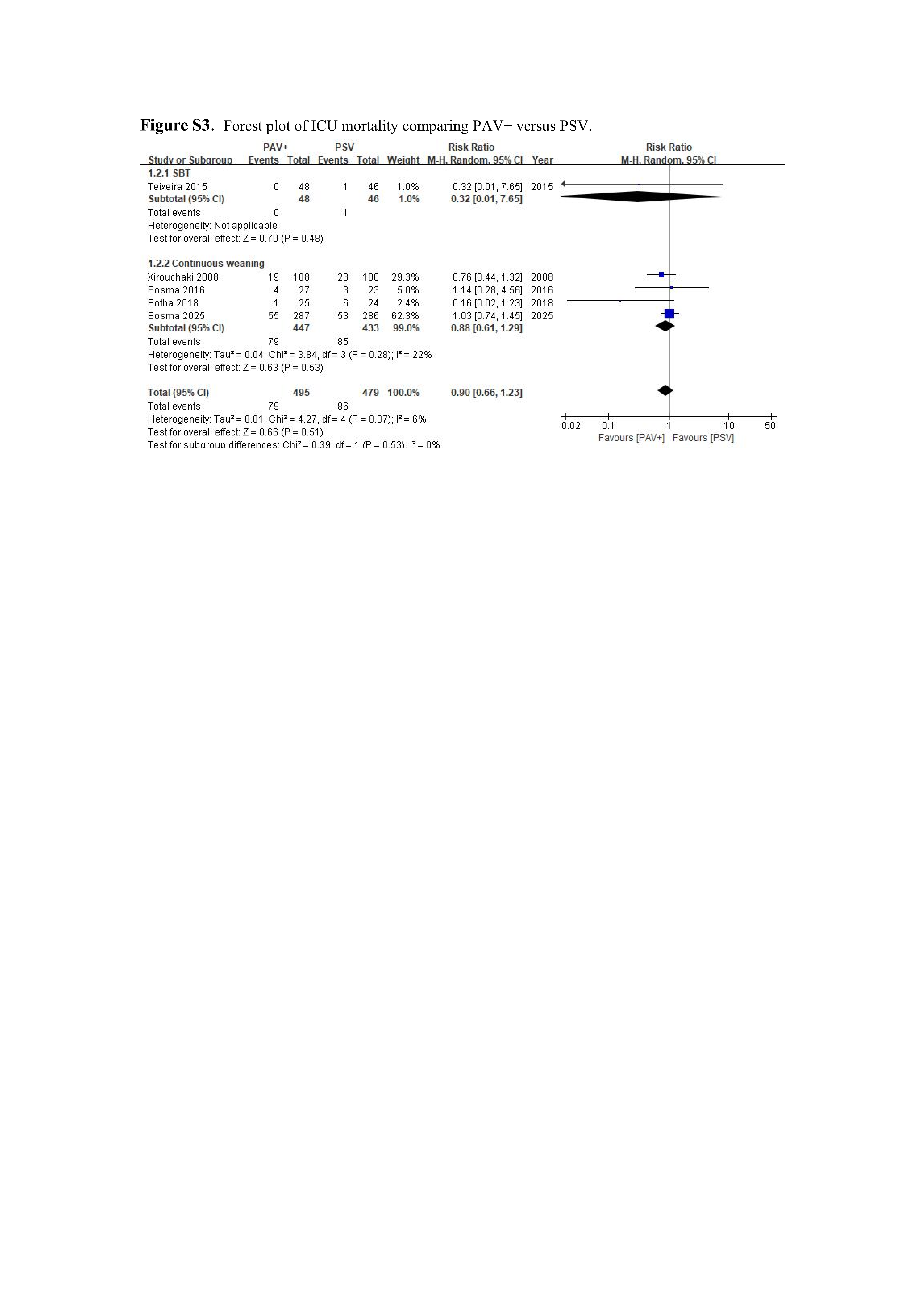

Supplement: Supplementary file 5 [file Image_3.png]

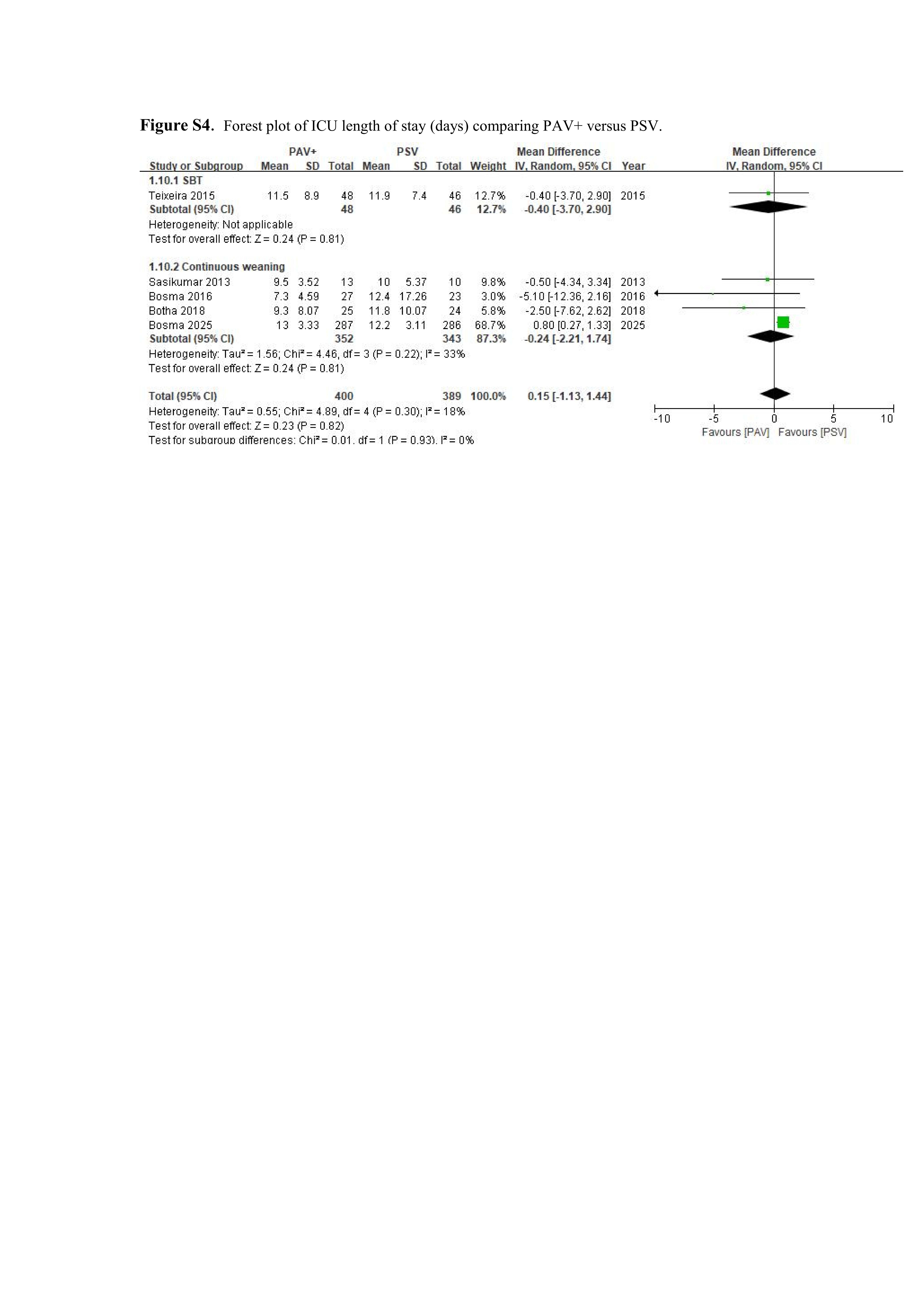

Supplement: Supplementary file 6 [file Image_4.png]

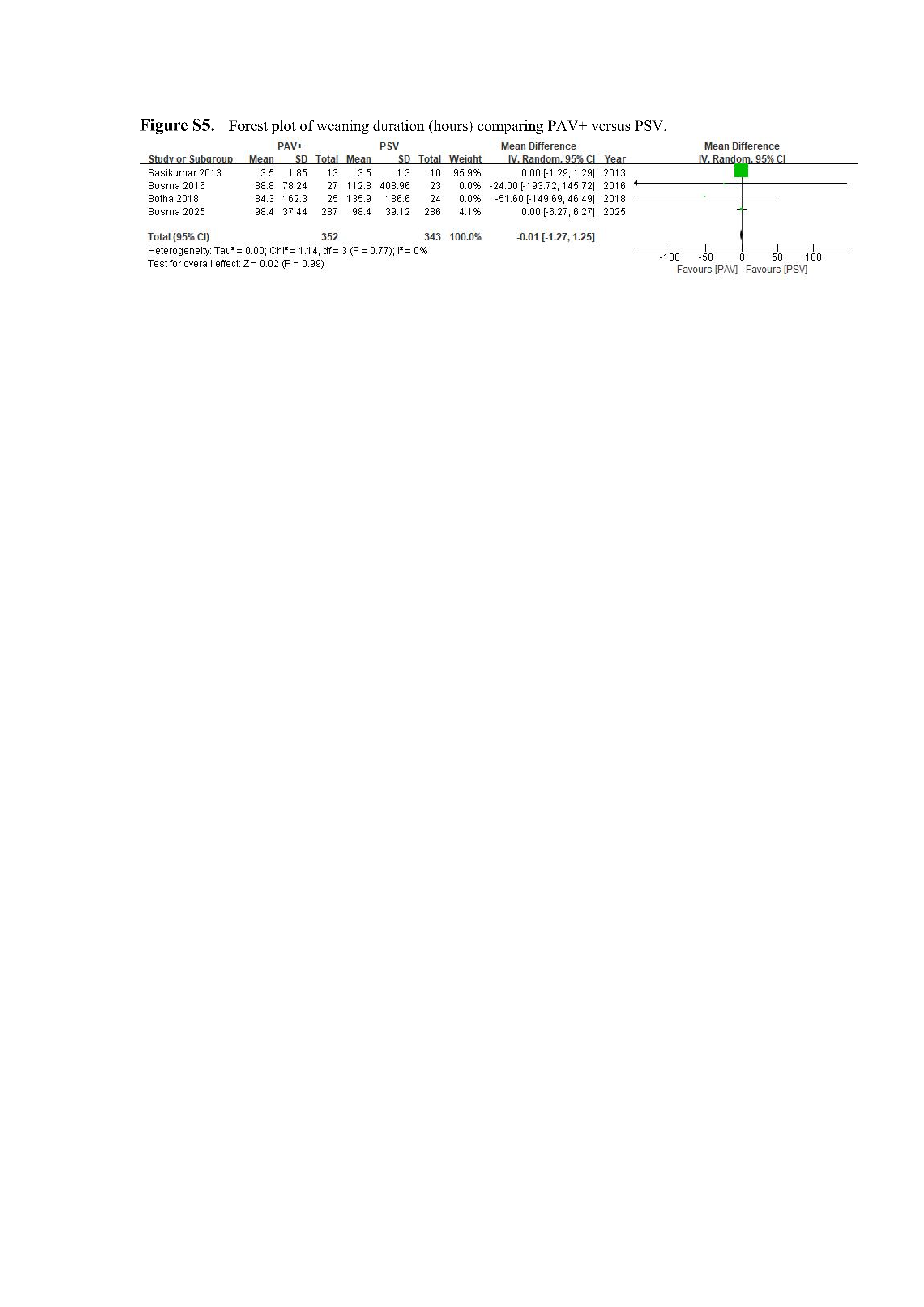

Supplement: Supplementary file 7 [file Image_5.png]
